# Activation of MAP Kinase Pathway by Polyisoprenylated Cysteinyl Amide Inhibitors Causes Apoptosis and Disrupts Breast Cancer Cell Invasion

**DOI:** 10.3390/biomedicines12030470

**Published:** 2024-02-20

**Authors:** Jassy Mary S. Lazarte, Nazarius S. Lamango

**Affiliations:** College of Pharmacy Pharmaceutical Sciences, Institute of Public Health, Florida A&M University, Tallahassee, FL 32307, USA; jassy.lazarte@famu.edu

**Keywords:** polyisoprenylated cysteinyl amide inhibitors (PCAIs), breast cancer, TNBC, mitogen-activated protein kinase (MAPK) signaling pathway, apoptosis, cell invasion

## Abstract

Prognoses for TNBC remain poor due to its aggressive nature and the lack of therapies that target its “drivers”. RASA1, a RAS-GAP or GTPase-activating protein whose activity inhibits RAS signaling, is downregulated in up to 77% of TNBC cases. As such, RAS proteins become hyperactive and similar in effect to mutant hyperactive RAS proteins with impaired GTPase activities. PCAIs are a novel class of agents designed to target and disrupt the activities of KRAS and other G-proteins that are hyperactive in various cancers. This study shows the anticancer mechanisms of the PCAIs in two breast cancer cell lines, MDA-MB-468 and MDA-MB-231. PCAIs (NSL-YHJ-2-27) treatment increased BRAF phosphorylation, whereas CRAF phosphorylation significantly decreased in both cell lines. Moreover, the PCAIs also stimulated the phosphorylation of MEK, ERK, and p90RSK by 116, 340, and 240% in MDA-MB-468 cells, respectively. However, in MDA-MB-231 cells, a significant increase of 105% was observed only in p90RSK phosphorylation. Opposing effects were observed for AKT phosphorylation, whereby an increase was detected in MDA-MB-468 cells and a decrease in MDA-MB-231 cells. The PCAIs also induced apoptosis, as observed in the increased pro-apoptotic protein BAK1, by 51%, after treatment. The proportion of live cells in PCAIs-treated spheroids decreased by 42 and 34% in MDA-MB-468 and MDA-MB-231 cells, respectively, which further explains the PCAIs-induced apoptosis. The movement of the cells through the Matrigel was also inhibited by 74% after PCAIs exposure, which could have been due to the depleted levels of F-actin and vinculin punctate, resulting in the shrinkage of the cells by 76%, thereby impeding cell movement. These results show promise for PCAIs as potential therapies for TNBC as they significantly inhibit the hallmark processes and pathways that promote cell proliferation, migration, and invasion, which result in poor prognoses for breast cancer patients.

## 1. Introduction

The effective management of triple-negative breast cancer (TNBC) has been a decades-long challenge due to its aggressive nature and, as the name implies, the lack of estrogen (ER) and progesterone receptors (PR) and human epidermal growth factor receptor type 2 (HER2) as drivers, for which there are targeted, more effective therapies [1]. TNBC accounts for approximately 15–20% of all newly diagnosed breast cancers, while accounting for 5% of all cancer-related deaths annually [2]. Apart from the conventional approach, which involves surgery and radiotherapy, the current common treatment for TNBC patients is chemotherapy [3]. Since TNBC has greater immunogenicity compared to other breast cancer subtypes, immuno-chemotherapy has been explored and shown to be efficacious in patients with PD-L1-positive tumors [4].

The emergence of targeted therapies such as Olaparib, Veliparib, and Erlotinib opened up a new horizon for TNBC treatment as these therapeutic strategies focus on the offending cancer-promoting molecular changes [5]. As previously extensively reviewed [6], numerous clinical trials have been conducted to determine the effectiveness of kinase inhibitors as treatments for human cancers driven by mutant kinases. The PI3K/AKT signaling pathway is aberrantly activated in breast cancer [7]. About 40% of hormone-receptor-positive (HR+) breast cancers cases are reported to harbor hyperactive mutant PI3K proteins [8], while only a small proportion occur in TNBC [9]. Although HR+ breast cancers respond positively to antihormonal therapies, it has been found that cancer progression in patients treated with these therapies often involves advanced-stage disease due to PI3K-dependent resistance mechanisms [10]. The use of targeted therapies in treating TNBC is necessary because of its heterogeneity, especially for patients who do not respond well to chemotherapy. Some of the proteins that have been the subject of targeted therapeutic development were identified by molecular profiling. These include poly-ADP ribose polymerase (PARP), phosphoinositide-3-kinase (PI3K), extracellular-signal-regulated kinase kinase (MEK), and protein kinase B (PKB or AKT) [11]. Because of the significant contribution of the PI3K/AKT signaling pathway to breast cancer progression, Pan-PI3K drugs targeting the four isoforms of class I PI3K were developed but showed significant toxicity [12]. Anti-PI3K drugs designed to specifically inhibit isoforms of class I PI3K still display side effects serious enough to terminate therapy in some cases [13]. PI3K inhibitors are most effective in patients in whose tumors the target enzyme is the mutant hyperactive isoform [14]. While *PI3KCA* mutations contribute only to about 8% of TNBC [14], PI3K/AKT inhibition is relevant to TNBC therapy due to the contribution of the pathway to resistance to TNBC chemotherapy [14]. Diminished activities of phosphatase enzymes such as PTEN contribute to overall phosphorylation activation of AKT.

The MAP kinase signaling pathway regulates processes involved in cell proliferation, differentiation, apoptosis, angiogenesis, and tumor metastasis [15]. Signaling through this pathway involves three main enzymes: MAP3K (RAFS), which phosphorylates and activates MAPKK (MAPK/ERK kinase, MEK), which then activates MAPK (extracellularly regulated kinase, ERK) [16]. It has been found that activating mutations of these kinases are deleterious as their hyperactivities result in diseases such as cancer [17]. Several inhibitors targeting these kinases have been approved and are currently in clinical use to treat various cancers. Although MAP kinase pathway activation underlies various neoplasms, excessive ERK stimulation has been widely reported to cause apoptosis. For example, cadmium treatment of HEK293 cells showed strong ERK activation and apoptosis [18]. ERK activation has also been observed in cells treated with anticancer agents [19] or NGF [20]. Furthermore, p90RSK substrates of ERK display either proliferative or apoptotic effects depending on which isoform is activated [21,22].

Approximately 78% of TNBC cases overexpress the human epidermal growth factor receptor type 1 (HER1/EGFR) [23], and up to 77% of TNBCs exhibit the downregulation of RASA1 [24]. Although the *KRAS* gene is aberrantly mutated such that the KRAS protein loses its intrinsic deactivating GTPase activity and thus drives only about 5% of breast cancer cases, the downregulation of RASA1, which is the GTPase-activating protein (GAP) that promotes KRAS deactivation through GTP hydrolysis [25,26], implies that KRAS is a significant contributor to cancer progression in 77% of TNBC. The receptor tyrosine kinase (RTK) HER2 (ErbB-2), a major target for breast cancer therapy, is overexpressed in about 25% of breast cancer cases [27]. Other RTKs, such as ErbB-3, IGF1R, and IGF2R, are overexpressed in 50–70, 50, and 40% of cases, respectively [28,29,30,31]. Since these growth-stimulating factors require KRAS for signaling, KRAS-targeting agents such as PCAIs have the potential to benefit a broad spectrum of breast cancer patients. The promise for such KRAS-targeting agents stems from PCAIs whose design is aimed at interfering with the polyisoprenylation-dependent interactions of KRAS and such related proteins as CDC42, RHOA, and RAC1. As shown for NSL-YHJ-2-27 in Table 1, the PCAIs are S-polyisoprenylated on the cysteine, as naturally occurs on the G-proteins. Instead of the carboxyl methylation that these G-proteins undergo, the carboxylic acid group of the cysteine in the PCAIs is amidated by reacting them with cycloalkyl amines to afford a biochemically more stable bioisostere. Furthermore, the α-amino group of the cysteine is coupled to moieties with ionizable groups such as the methyl-piperazine, as in NSL-YHJ-2-27. Although these ionizable groups were originally intended to promote the aqueous solubilities of the otherwise very hydrophobic PCAIs [32], we later found that the positive charges thus formed contributed significantly to the anticancer activities of the PCAIs [33]. These agents were designed to inhibit polyisoprenylated methylated protein methyl esterase (PMPMEase), which we found to be overexpressed in various cancers [32,34,35]. The PCAIs showed only minimal PMPMEase inhibition, with their potencies against cancer cell viability surpassing the enzyme-inhibitory activities [32], thus indicating a pharmacological target that is different from PMPMEase. PCAIs have been shown to suppress cell migration, invasion, and growth [36]; induce caspase-dependent apoptosis [37]; and activate MAPK pathway kinases in various lung and pancreatic cancer cell lines [33]. Here, we determined the effects of the PCAIs on the phosphorylation of the RAF/MEK/ERK kinases, examined their role in inducing apoptosis, and, finally, evaluated the PCAIs’ ability to inhibit the growth, migration, and invasion; the cytoskeleton; and the focal adhesion and vinculin in breast cancer cells.

## 2. Materials and Methods

### 2.1. Materials

Cell lines were purchased from the American Type Culture Collection (ATCC, Manassas, VA, USA). Primary antibodies specific to phosphorylated proteins BRAF (p-BRAF, Cat #2696), CRAF (p-CRAF, Cat #9427), MEK 1/2 (p-MEK 1/2, Cat #9154S), ERK 1/2 (p-ERK1/2, Cat #4370S), p90RSK (p-p90RSK, Cat #11989S), AKT (p-AKT, Cat # 4060s), vinculin (E1E9V, Cat #13901), full-length Caspase 3 (Cat #9662S), full-length Caspase 7 (Cat #9492S), secondary antibodies anti-mouse IgG, HRP-linked antibody (Cat #7076), and anti-rabbit IgG, HRP-linked antibody (Cat #7074), and housekeeping protein GAPDH (Cat #5174S) or α-actinin (Cat #6478S) were purchased from Cell Signaling Technology (Danvers, MA, USA). The PCAIs used in this study (Table 1) were synthesized in our lab as previously described [33]. NSL-YHJ-2-62 was used as a negative control. It lacks the polyisoprenyl moiety, which is one of the most important components of the PCAIs pharmacophore.

### 2.2. Cell Culture

MDA-MB-231 and MDA-MB-468 cells were cultured in high-glucose Modified Eagle Medium (DMEM) (Genesee Scientific, San Diego, CA, USA). The media were supplemented with 10% heat-inactivated fetal bovine serum (FBS), 100 U/mL penicillin, and 100 μg/mL streptomycin (Genesee Scientific, San Diego, CA, USA). The cells were cultured at 37 °C in 5% CO_2_/95% humidified air. In all experiments, the cells were grown in experimental medium, which was the base medium supplemented with 5% heat-inactivated fetal bovine serum.

### 2.3. Effects of PCAIs on the Viability of Breast Cancer Cells

To determine the effect of the PCAIs on the viability of MDA-MB-231 and MDA-MB-468 cells, cell viability assays were conducted. Briefly, respective breast cancer cell lines were plated into 96-well culture plates (Genesee Scientific, San Diego, CA, USA) at a density of 1 × 10^4^ cells/well in the experimental medium. The cells were allowed for 24 h to adhere. The cells were treated with varying concentrations of PCAIs (0.5, 1, 2, 5, 10, 20, and 50 µM). Control cells were treated with 1% acetone in the experimental medium. Treatments were repeated after 24 h for the 48 h exposure. To determine the relative viabilities of the treated cells, the resazurin reduction assay was conducted, in which 0.02% of resazurin reagent was added to the wells. The plates were incubated at 37 °C in 5% CO_2_/95% humidified air for 1.5 h. Thereafter, fluorescence intensities were determined with excitation at 544 nm and emission at 590 nm using SoftMax Pro Reader version 5.4 for Windows (Molecular Devices, San Jose, CA, USA). The cell viability results were expressed as percentages of fluorescence in the treated cells relative to the control (0 μM). To obtain the EC_50_ values, the fluorescence intensities were plotted against concentrations in non-linear regression curve fits using GraphPad Prism version 8.0 for Windows (San Diego, CA, USA).

### 2.4. PCAIs’ Inhibition of Cell Proliferation

Here, we determined whether the PCAIs affected the ability of the breast cancer cells to proliferate. MDA-MB-231 and MDA-MB-468 cells (2 × 10^5^ cells/well) were seeded into 21.2 cm^2^ tissue culture dishes (Genesee Scientific, San Diego, CA, USA). The baseline number of cells was determined by counting the cells from a designated dish. The other remaining dishes with adherent cells were treated with the respective concentrations of NSL-YHJ-2-27 for 48 h. Using a Countess II automated cell counter (Life Technology Corporation, Grand Island, NY, USA), viable cells were recorded from three independent experiments. The number of proliferated cells was obtained by subtracting the initial number of cells immediately before treatment from the final number of cells after 48 h exposure. The data were plotted using GraphPad Prism version 8.0 for Windows (San Diego, CA, USA).

### 2.5. Effects of Long-Term Treatment of PCAIs on Breast Cancer Cells

To determine the effect of long-term PCAIs treatment on the cells, 1 × 10^5^ cells were seeded onto a 12-well plate. Upon adherence, triplicate sets were treated daily with 0, 1, 2, and 5 μM of PCAIs. Images were captured daily using a Nikon Ti Eclipse microscope (Nikon Instruments Inc. Melville, NY, USA) at 10× magnification for analysis. Treatment was stopped when 100% of the cells were judged to be non-viable. 

### 2.6. Effects of PCAIs on the Phosphorylation of MAPK and PI3K/AKT Pathway Enzymes

To examine the effects of the PCAIs on the activation of the MAPK and PI3K/AKT pathway, Western blot analysis was performed. The cells were plated into 60.8 cm^2^ tissue culture dishes (Genesee Scientific, San Diego, CA, USA) in experimental medium at a cell density of 1 × 10^6^ cells/dish and left for 24 h to adhere. The cells were then supplemented with fresh experimental medium, followed by treatment with varying concentrations of the PCAIs NSL-YHJ-2-27 and control analog NSL-YHJ-2-62. Treatments were repeated after 24 h. At 48 h from the onset of treatment, the experimental media were removed and the cells washed thrice with 1x PBS. Cell lysis was performed by incubating the cells in RIPA lysis buffer supplemented with 0.1% *v*/*v* protease/phosphatase inhibitor cocktail (Cell Signaling Technology, Danvers, MA, USA). Protein quantification on the lysates was performed using the Quick Start™ Bradford protein assay (Bio-Rad, Hercules, CA, USA).

### 2.7. Western Blot Assay

Equal amounts of proteins (30 μg) were mixed with XT sample buffer and XT reducing agent (Bio-Rad, Hercules, CA, USA) and denatured with boiling water for 5 min. Separation of the proteins was performed by SDS-PAGE using 4–12% Criterion™ XT Bis-Tris protein gradient gels, after which the proteins were transferred onto Trans-Blot turbo midi 0.2 µm nitrocellulose membranes (Bio-Rad, Hercules, CA, USA). The membranes were blocked using OneBlock™ western-CL blocking buffer (Genesee Scientific, San Diego, CA, USA) for 1 h at room temperature. The membranes were then treated with the respective primary antibodies targeting the phosphoproteins by shaking at 4 °C overnight. These were then washed thrice with 1x TBST (Genesee Scientific, San Diego, CA, USA) and incubated at room temperature with either anti-rabbit or anti-mouse IgG HRP-linked antibodies for 1 h. The membranes were immersed in Radiance Plus (Azure Biosystems, Dublin, CA, USA) ECL reagents, following the manufacturer’s recommendations, and imaged using the ChemiDoc XRS+ System (Bio-Rad, Hercules, CA, USA). Phosphorylation levels were determined by quantifying the chemiluminescent intensities using Image Lab 6.0 (Bio-Rad, Hercules, CA, USA) and normalizing against the corresponding GAPDH band intensities. Three independent experiments were conducted, and the protein levels were plotted using GraphPad Prism version 8.0 for Windows (San Diego, CA, USA).

### 2.8. Apoptotic Effects of PCAIs on MDA-MB-468

The mechanism of the PCAIs-induced cell death was determined by evaluating two important apoptotic markers, Caspases 3 and 7, using Chemicon’s CaspaTag In Situ Caspase Detection Kit (Millipore Corp, Burlington MA, USA), followed by flow cytometry analysis. Specifically, cells were plated in 60.8 cm^2^ tissue culture dishes (Genesee Scientific, San Diego, CA, USA) at a density of 5 × 10^5^ cells/dish and grown overnight in experimental medium. The adherent cells were treated with varying concentrations of NSL-YHJ-2-27 at the onset and again after 24 h. After 48 h, the cells were harvested using 1x PBS suspended in experimental media and transferred to new sterile tubes. The labeling of the cells was affected by gently mixing 30x FLICA reagent into the suspension, followed by incubation for 1 h at 37 °C under 5% CO_2_ while protecting the tubes from light. The cells were washed twice with 1x wash buffer and centrifuged at 500× *g* for 5 min at room temperature. The cells were then resuspended in 1x wash buffer. Propidium iodide was added into the final suspension to label the dead cells. Cells were then analyzed using Sony SH800 Cell Sorter Software version 2.1.6 (Sony Biotechnology, San Jose, CA, USA). In addition to Caspase 3 and 7, a pro-apoptotic protein, BCL2-antagonist killer 1 (BAK1), was evaluated to determine the apoptotic effects of NSL-YHJ-2-27 by Western blotting. Cells (1 × 10^6^ cells/dish) were seeded into 60.8 cm^2^ tissue culture dishes (Genesee Scientific, San Diego, CA, USA) and allowed overnight to adhere. Varying concentrations of the PCAIs (NSL-YHJ-2-27) were used to treat the cells for 48 h. The preparation of cell lysates and Western blot analysis was performed as described in Section 2.6 and Section 2.7.

### 2.9. Effects of PCAIs on 3D Spheroids

To determine the effects of NSL-YHJ-2-27 on a 3D spheroid culture, both breast cancer cell lines (5 × 10^3^ cells/well) were seeded into 96U round bottom Nunclon Sphera Plates and incubated for 48 h for the spheroids to form. This was followed by PCAIs treatment at 24 and 48 h. Three replicates were prepared for each treatment concentration. On the third day post-treatment, the spheroids were stained with 5 µg/mL of an acridine orange/ethidium bromide (AO/EB) solution. The spheroids were observed for 72 h and images captured using a Nikon Ti Eclipse microscope at 4× magnification at 0 and 72 h. Using the NIS Element software version 4.30.02 (Nikon Instruments Inc. Melville, NY, USA), the ratio of the fluorescent intensities of AO over EB for each PCAIs treatment concentration was computed and plotted using GraphPad Prism version 8.0 for Windows (San Diego, CA, USA).

### 2.10. Effects of Inhibitors of Various RSK Isoforms on PCAsI-Induced Cell Death

The activation of some p90RSKs has been reported to induce apoptosis. To determine whether RSK inhibitors might reverse the PCAIs’ effects on cell viability, an RSK inhibitor that blocked the action of RSK 1, RSK 2, and RSK 3 was used in cell viability assays, in a similar fashion as described in Section 2.3, but specifically varying the concentrations of PCAIs and RSK 1/2/3 inhibitors in quadruplicate as follows: (a) PCAIs (NSL-YHJ-2-27) only, (b) RSK1/2/3 only, (c) NSL-YHJ-2-27 and 1.5 µM RSK1/2/3 to inhibit RSK 1, (d) NSL-YHJ-2-27 and 2.4 µM RSK1/2/3 to inhibit RSK 2, and (e) NSL-YHJ-2-27 and 1.2 µM RSK1/2/3 to inhibit RSK 3 [38]. Treatments were performed for 48 h, followed by cell viability analysis using the resazurin reduction assay, as indicated earlier. 

### 2.11. Effects of PCAIs on Breast Cancer Cell Invasion

The effect of the PCAIs on cell invasion was determined in Matrigel. MDA-MB-231 cells (5 × 10^3^ cells/well) were seeded into a 96U round bottom Nunclon Sphera Plate in 200 μL complete medium and allowed for 48 h to obtain compact spheroids. Half of the complete medium was removed and the spheroids treated with the respective concentrations of NSL-YHJ-2-27. In a separate prechilled tube, 30% BD Matrigel (Corning, Bedford, MA, USA) was thoroughly and gently mixed with twice the respective concentrations of NSL-YHJ-2-27. Using a prechilled tip, the Matrigel–PCAIs mixture was carefully dispensed into the wells containing the spheroids. The spheroids were left to grow in the incubator for 72 h. The Matrigel-embedded spheroids were monitored for invading cells, with images taken at 0 and 72 h using a Nikon Ti Eclipse microscope at 4× magnification. The invasion areas were quantified using the NIS Element software version 4.30.02 (Nikon Instruments Inc. Melville, NY, USA) and then plotted and analyzed using GraphPad Prism version 8.0 for Windows (San Diego, CA, USA).

### 2.12. Effects of the PCAIs on Actin Filaments and Vinculin Punctates

F-actin assembly and organization are important for both cell migration and adhesion. Here, we evaluated the effect of the PCAIs on actin filaments and vinculin. To do this, a cell suspension containing 6 × 10^3^ cells was seeded onto an 8-well i*bidi* µ-slide and allowed for 24 h to adhere, followed by exposure to NSL-YHJ-2-27 for 48 h. The cells were then fixed with 4% paraformaldehyde, permeabilized using 0.5% Triton X-100, and stained with Alexa Fluor 568 Phalloidin and Hoechst. The slide was visualized using a Keyence BZ-X800 fluorescence microscope (Keyence Corporation of America, Itasca, IL, USA) at 20× magnification. Fluorescence intensity per cell was determined for 300 cells per treatment. Data were analyzed and plotted using GraphPad Prism version 8.0 for Windows (San Diego, CA, USA). Furthermore, to determine whether the PCAIs affected focal adhesion, the effects on vinculin in MDA-MB-468 cells were determined. After PCAIs treatment, the cells were fixed, permeabilized, and incubated with 1% BSA solution for 1 h, followed by incubation overnight in 1% BSA mixed with anti-vinculin antibodies (Cell Signaling Technology, E1E9V). Thereafter, the cells were incubated overnight in anti-rabbit IgG Alexa Fluor 555 conjugate (Cell Signaling Technology, MA, USA). The slides were visualized using a Keyence BZ-X800 fluorescence microscope at 40× magnification. To determine whether the vinculin levels were affected by PCAIs treatment, Western blot analysis was performed. Cells were prepared and treated with PCAIs as in Section 2.6. The preparation of cell lysates and Western blot analysis were performed as described in Section 2.7, and they were probed for vinculin using anti-vinculin monoclonal antibodies. 

### 2.13. Statistical Analysis

All results are the means ± SEM. To determine statistical significance, the values of the treatment group were compared to the respective controls either by one-way ANOVA with Dunnett’s post-hoc tests or two-way ANOVA with Dunnett’s multiple comparisons tests, using GraphPad Prism version 8.0 for Windows, and *, *p* < 0.05; **, *p* < 0.01; ***, *p* < 0.001; ****, *p* < 0.0001 were considered significant.

## 3. Results

### 3.1. PCAIs Suppress Breast Cancer Cell Viability

**Figure 1:**
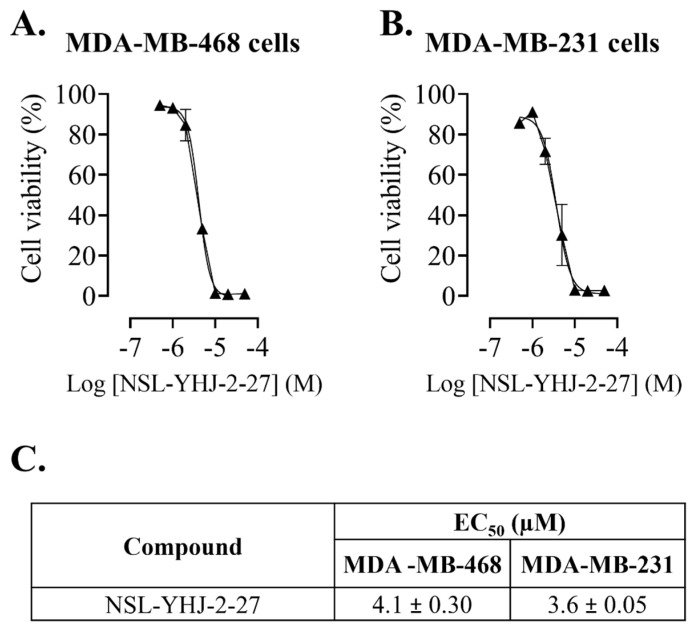
NSL-YHJ-2-27 suppresses the viability of breast cancer cells. After treatment of cells with the indicated concentrations of NSL-YHJ-2-27 (0, 0.5, 1, 2, 5, 10, 20, and 50 µM), resazurin reduction assay was performed to determine the viability of the cells. (**A**,**B**) Cell viabilities were plotted against varying concentrations of NSL-YHJ-2-27 expressed as the percentage of fluorescence intensity compared to the controls. (**C**) EC_50_ values for the effects of the treatments on cell viability.

The potency of the PCAIs against the breast cancer cell lines was determined (Figure 1A,B). The results reveal the high potency of NSL-YHJ-2-27, with EC_50_ values of 4.1 ± 0.30 and 3.6 ± 0.05 µM, in MDA-MB-468 and MDA-MB-231 cells, respectively (Figure 1C).

### 3.2. PCAIs Inhibit the Proliferation of Breast Cancer Cells

**Figure 2:**
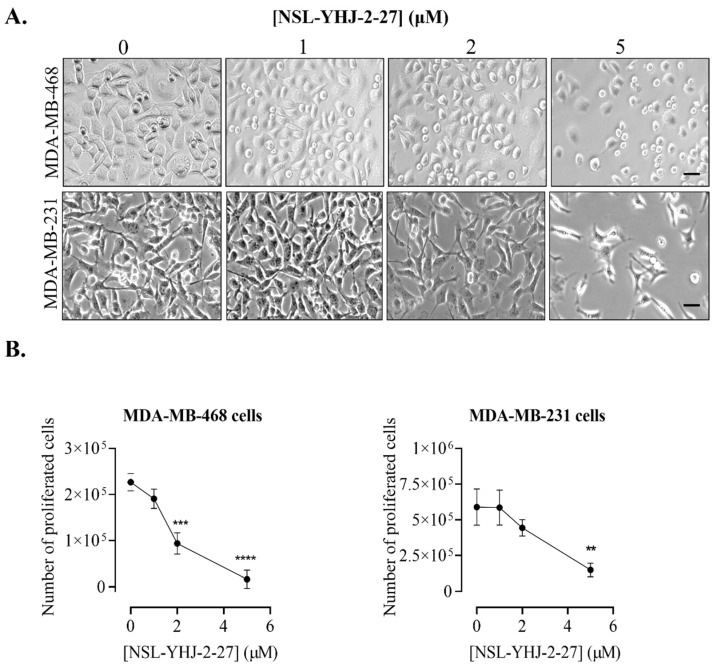
NSL-YHJ-2-27 blocks proliferation and induces cell rounding and death of breast cancer cells. Cells were seeded onto six-well plates and then treated with the indicated concentrations (0, 1, 2, and 5 µM) of PCAIs for 48 h. (**A**) Images of the cells were then captured using Nikon Ti Eclipse microscope at 10× magnification. Scale bar = 100 µm. (**B**) The number of cells that proliferated after PCAI treatment was obtained by subtracting the final number of cells from the initial count of cells before PCAI treatment. Significance was calculated from three independent experiments. Statistical significance (**, *p* < 0.01; ***, *p* < 0.001; ****, *p* < 0.0001) was determined by one-way ANOVA with post hoc Dunnett’s test.

The inhibition of the proliferative cell growth that may develop into tumors is an essential criterion in the development of cancer therapies. Concurrent with the cell viability result, breast cancer cells treated with PCAIs became rounded, ultimately leading to death, as shown in Figure 2A. More importantly, the number of proliferated cells decreased as the concentration of PCAIs increased. In MDA-MB-468 cells, 16 ± 7.1% inhibition of proliferation was observed after treatment with 1 μM PCAIs. Higher PCAIs concentrations caused the number of proliferated cells to decrease by 59 ± 5 and 93 ± 3.5% in cells treated with 2 μM and 5 μM PCAIs, respectively (Figure 2B). Moreover, MDA-MB-231 cells also showed significant decreases in proliferated cells by 52 ± 5.5 and 75 ± 5.5% after 2 μM and 5 μM PCAIs treatment, respectively (Figure 2B). 

### 3.3. Long-Term Treatment of NSL-YHJ-2-27 Results in Cell Death

**Figure 3:**
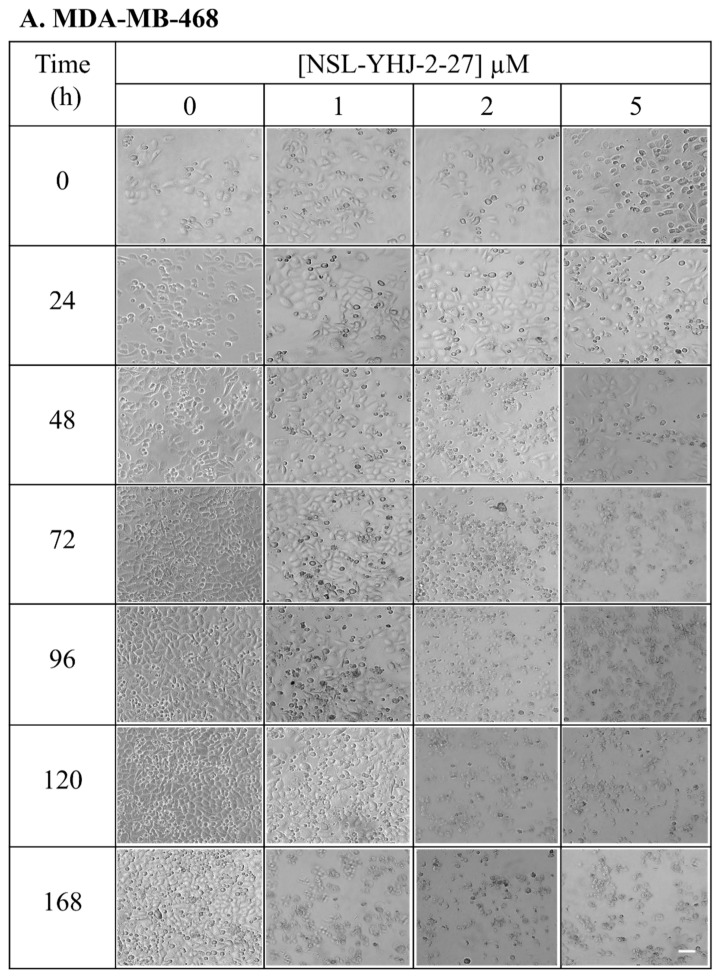

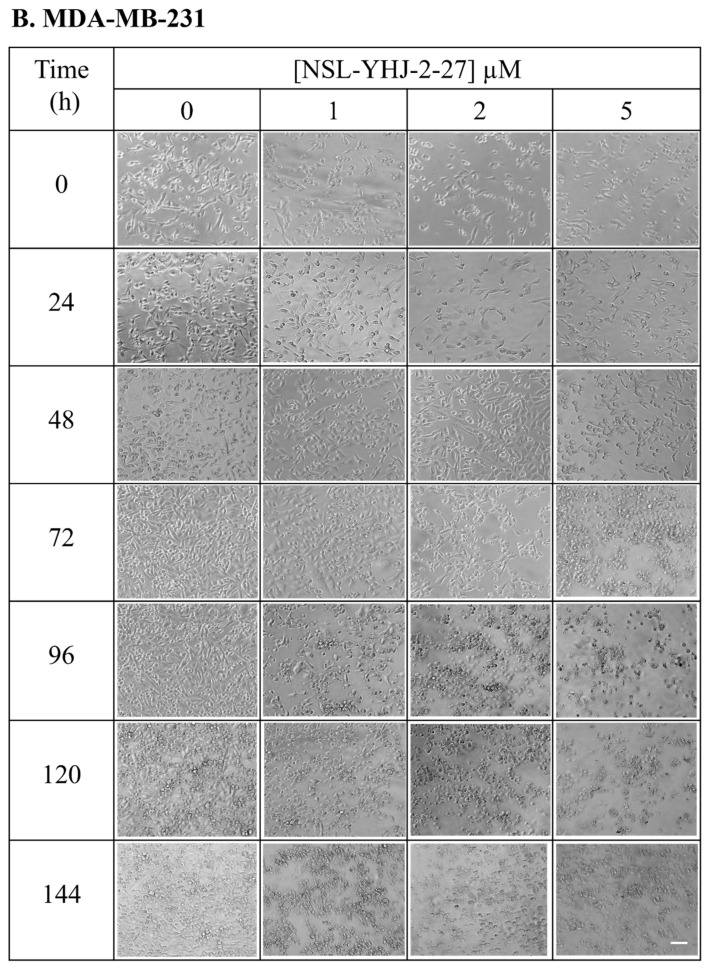
Breast cancer cells do not survive long-term PCAIs treatment. Cell suspensions consisting of 100,000 cells were seeded onto twelve-well plates and exposed to the indicated concentrations of PCAIs (0, 1, 2, and 5 µM). Images were captured daily at 10× magnification using Nikon Ti Eclipse microscope. Scale bar = 100 µm.

Repeated treatment with PCAIs resulted in the eventual death of all the cells. Evident cell death was first observed in cells that were treated with 5 µM PCAIs rather than those treated with lower concentrations (1–2 µM PCAIs). In MDA-MB-468 cells, the rounding of the cells was first observed 24 h after treatment with 5 µM PCAIs, culminating in no surviving cells after 72 h. It was followed by cells treated with 2 µM PCAIs, which all died by the 96th hour of treatment. Lastly, the cells treated with the lowest concentration of PCAIs (1 µM) survived until the 168th hour. On the other hand, untreated cells were alive throughout (Figure 3A). In the case of MDA-MB-231 cells, the first sign of cell rounding was observed at 48 h in cells exposed to 5 µM PCAIs, culminating in total cell death by 72 h. Cell rounding became prominent in cells treated with 2 µM PCAIs after 72 h, with total cell death by the 96th hour. In cells exposed to 1 µM PCAIs, the presence of rounded cells was observed after 96 h and total cell death by the 144th hour, while the control cells were still alive (Figure 3B).

### 3.4. PCAIs Stimulate the Phosphorylation of MAPK Pathway Enzymes

**Figure 4:**
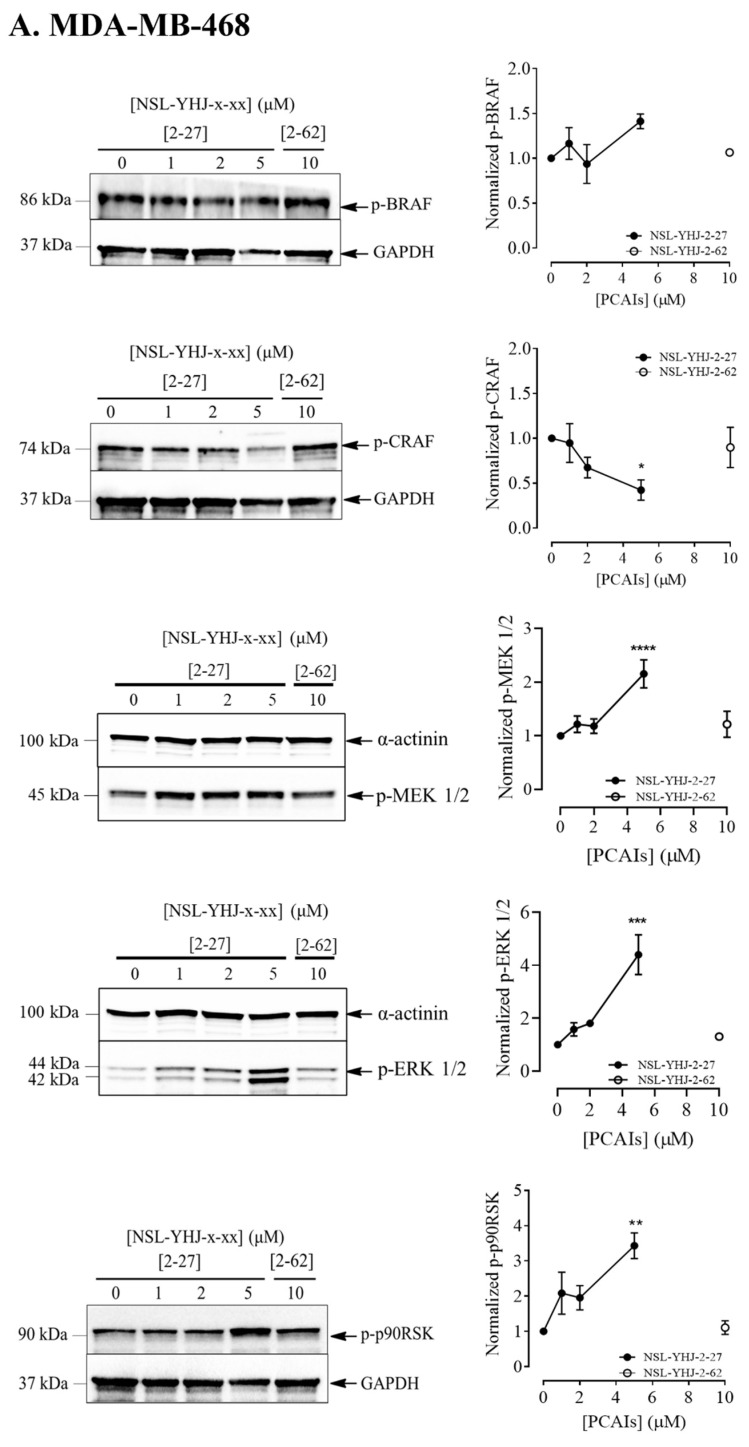

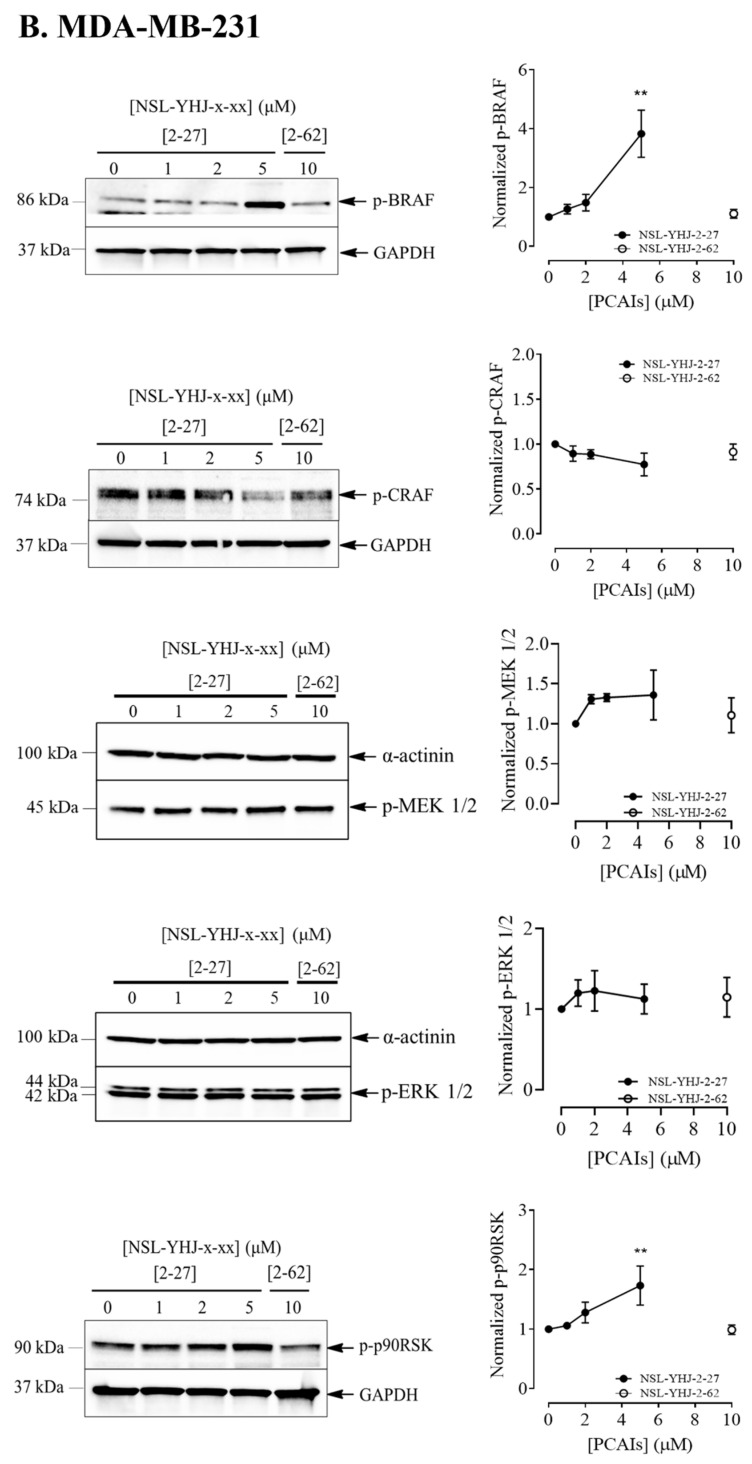
NSL-YHJ-2-27 exerts pronounced effects on the phosphorylation of MAPK pathway enzymes. Cells were treated with the indicated concentrations of NSL-YHJ-2-27 (0, 1, 2, and 5 µM) or the non-farnesylated analog NSL-YHJ-2-62 (10 µM) for 48 h. They were then lysed and analyzed by Western blotting as described in the Methods section. Data are representative of three independent experiments. Statistical significance (*, *p* < 0.05; **, *p* < 0.01; ***, *p* < 0.001; **** *p* < 0.0001) was determined by one-way ANOVA with post hoc Dunnett’s test.

MAPK pathway intermediates typically undergo phosphorylation-mediated activation. Given the role of MAPK pathway signaling in cell proliferation, we evaluated the effects of the PCAIs on their phosphorylation. Phosphorylated BRAF increased by 41 ± 3.8 and 280 ± 7.4% in MDA-MB-468 and MDA-MB-231 cells, respectively, while the phosphorylation of CRAF significantly decreased by 58 ± 11.3% and 23 ± 12.6% in MDA-MB-468 and MDA-MB-231 cells, respectively. MEK 1/2 phosphorylation significantly increased by 116 ± 3.2% and 36 ± 18% in MDA-MB-468 and MDA-MB-231 cells, respectively. While ERK 1/2 phosphorylation increased by 340 ± 5.4% in MDA-MB-468, there was no apparent ERK1/2 phosphorylation in MDA-MB-231 at the indicated time point following PCAIs treatment. Lastly, we evaluated the level of phosphorylation of one of the substrates of ERK1/2, p90RSK, and found that p90RSK phosphorylation increased by 240 ± 3.3% in MDA-MB-468 cells and 73 ± 15% in MDA-MB-231 cells (Figure 4A,B). The results obtained indicate a pronounced effect of the PCAIs on the MAPK pathway.

### 3.5. PCAIs’ Effects on AKT Pathway May Have Caused Cell Death in KRAS-Mutant Breast Cancer Cells

**Figure 5:**
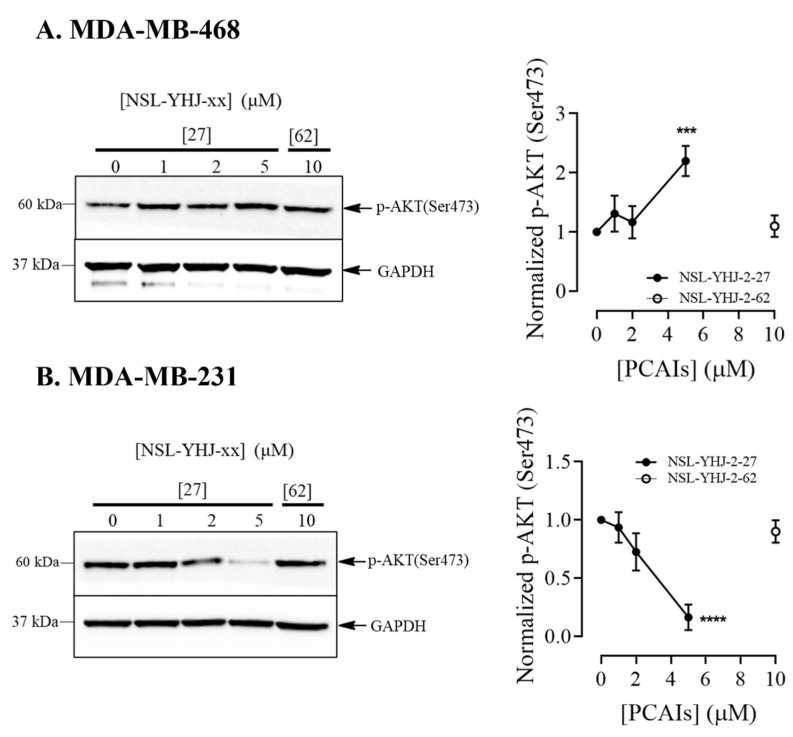
NSL-YHJ-2-27 reduces AKT phosphorylation in KRAS-mutant breast cancer cells. (**A**,**B**) Cells were treated with the indicated concentrations of PCAIs NSL-YHJ-2-27 (0, 1, 2, and 5 µM) or NSL-YHJ-2-62 (10 µM) and analyzed for AKT phosphorylation levels by Western blotting, as described in the Methods. Data are representative of three independent experiments. Statistical significance (***, *p* < 0.001, ****, *p* < 0.0001) was determined by one-way ANOVA with post hoc Dunnett’s test.

Another important pathway that is implicated in the progression of breast cancer is the PI3K/AKT pathway, which is involved in the regulation of many cellular processes, including cell growth, proliferation, and apoptosis [39,40]. Cross-talk between the MAPK and PI3K/AKT pathways in response to various stimuli has been reported to modify the signaling intensities and responses [41]. Here, we determined the effect of PCAIs on AKT phosphorylation in the two breast cancer cell lines. In MDA-MB-468 cells, a 120 ± 0.15% increase in AKT phosphorylation was observed (Figure 5A). Meanwhile, in MDA-MB-231 cells, a prominent decrease of 84 ± 0.06% was observed (Figure 5B). While it is tempting to assume that the mutant KRAS proteins may account for the differences in AKT phosphorylation in response to PCAIs treatment, more studies need to be performed with more cell lines to make such a conclusion, since the molecular differences between the two cell lines used in the current study may not be defined solely by their *KRAS* mutation status. 

### 3.6. PCAIs Induce Cell Death by Apoptosis

**Figure 6:**
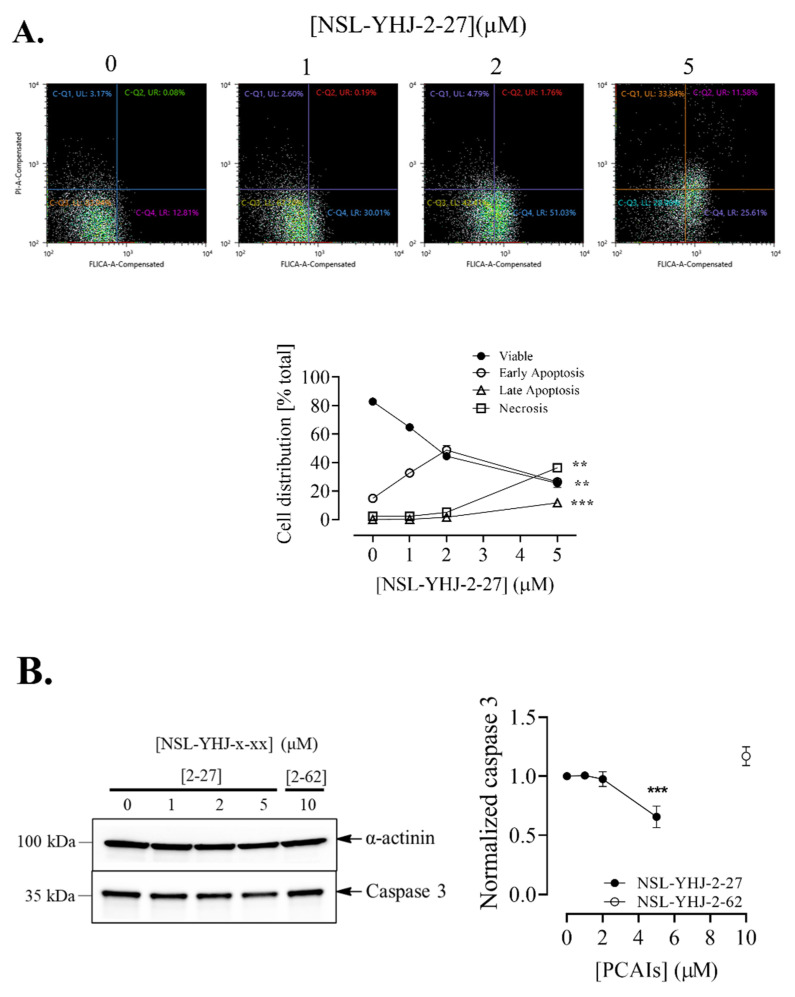

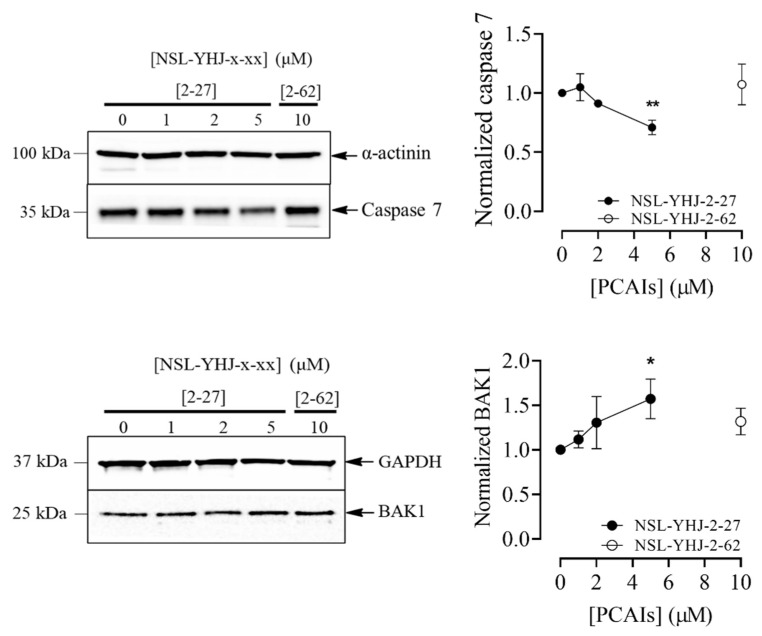
NSL-YHJ-2-27 induces apoptosis in MDA-MB-468 cells. (**A**) Cells were treated with the indicated concentrations of NSL-YHJ-2-27 (0, 1, 2, and 5 µM), collected, and stained with FLICA and propidium iodide, followed by flow cytometry analysis. (**B**) PCAIs-treated cells, NSL-YHJ-2-27 (0, 1, 2, and 5 µM) or NSL-YHJ-2-62 (10 µM), were analyzed for apoptotic factors by Western blotting, as described in the Methods. Data are representative of three independent experiments. Statistical significance (*, *p* < 0.05; **, *p* < 0.01; ***, *p* < 0.001) was determined by one-way ANOVA with post hoc Dunnett’s test.

The ability of a compound to induce cell death in cancer cells is an essential characteristic of anticancer therapies. As the number of dead cells were very noticeable after treatment with 5 µM of NSL-YHJ-2-27, we evaluated the effect of PCAIs on MDA-MB-468 by determining the levels of Caspases 3 and 7, which are markers of apoptosis, using flow cytometry analysis. The flow cytometry results revealed a concentration-dependent increase in the levels of Caspases 3/7 in the cells. Treatment with 1 and 2 µM NSL-YHJ-2-27 caused a 30 ± 1.47 and 51 ± 5% shift in the cell population from viable to early apoptosis, while 5 µM PCAIs resulted in 26 ± 0.6, 12 ± 0.07 and 34 ± 1.5% of the cells in early and late apoptosis and necrosis, respectively (Figure 6A). Furthermore, Western blotting analysis revealed significant decreases in the levels of full-length Caspases 3 and 7, by 34 ± 5.3 and 29 ± 3.3%, respectively, while the levels of the pro-apoptotic protein BAK1 (Bcl2-antagonist killer 1) increased by 51 ± 1.8% (Figure 6B).

### 3.7. PCAIs Treatment Disaggregates Compact 3D Spheroids

**Figure 7:**
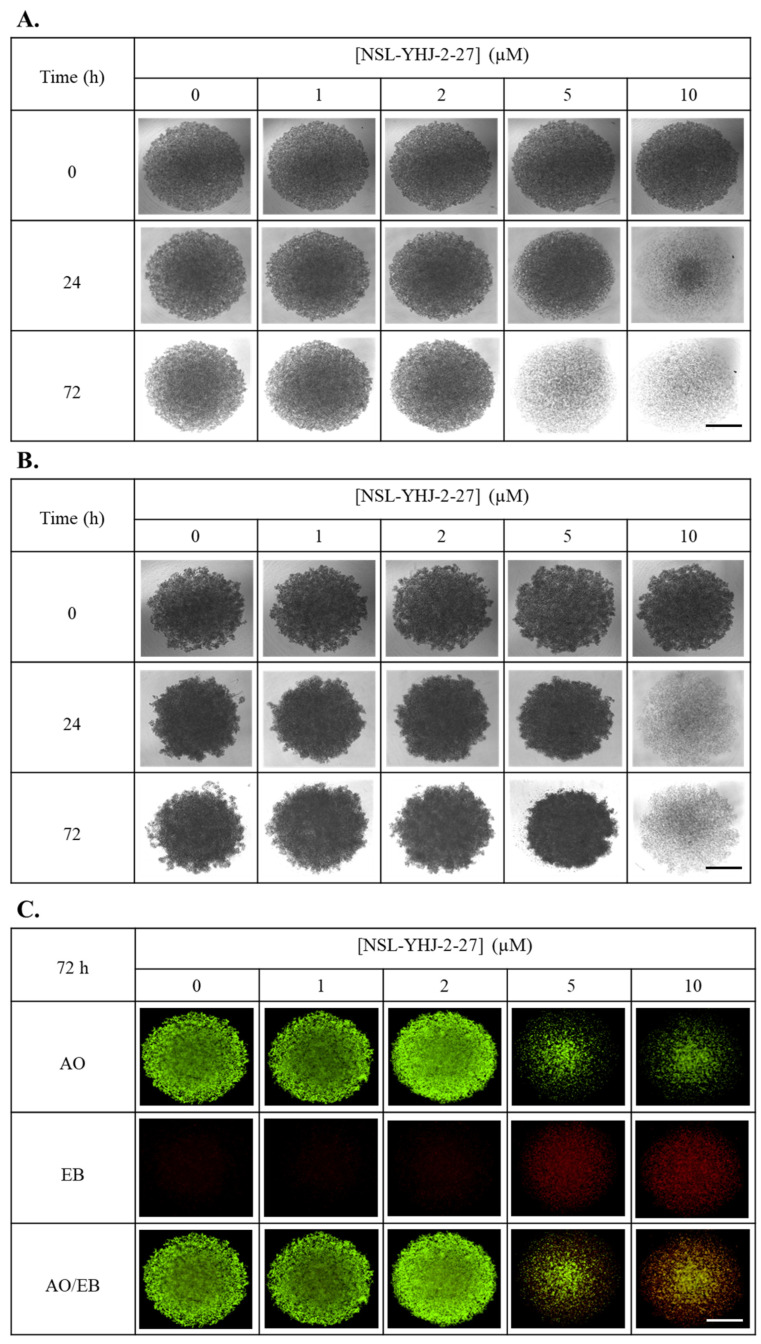

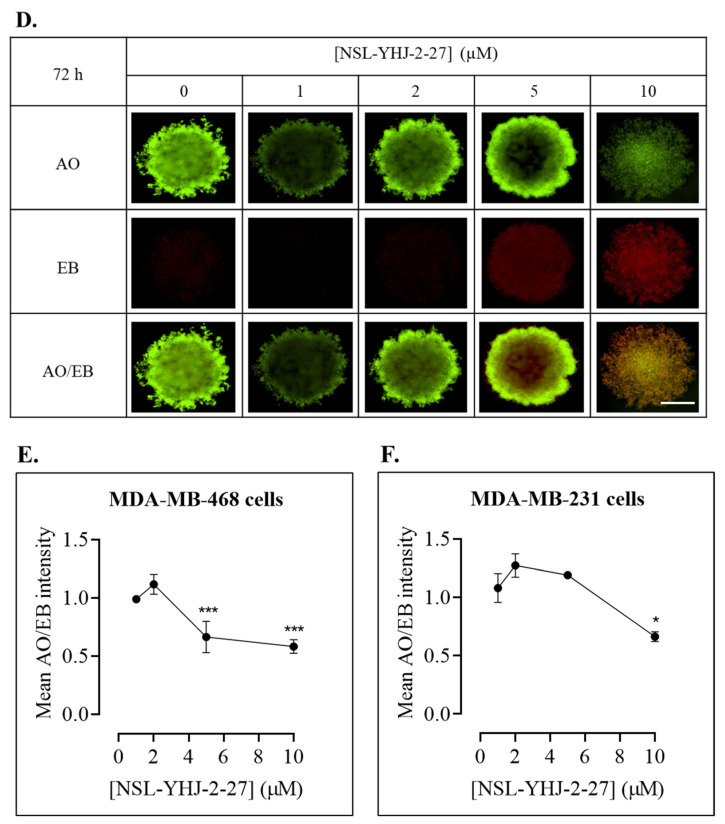
NSL-YHJ-2-27 treatment disaggregates compact spheroids. MDA-MB-468 and MDA-MB-231 cells were seeded in 96U round bottom Nunclon Sphera Plate and allowed to grow for 48 h. Once compact spheroids were formed, they were treated with the indicated concentrations of NSL-YHJ-2-27 (0, 1, 2, 5, and 10 µM) and observed for 72 h. They were then stained with 10 µg/mL of AO/EB dye mixture at 72 h post-PCAIs-treatment and brightfield (BF) (**A**,**B**) and fluorescent (**C**,**D**) images were captured at 4× magnification using a Nikon Ti Eclipse microscope. Scale bar = 100 µm. The mean intensity ratios AO/EB equivalent to the viable-to-dead cells in the spheroids were determined (**E**,**F**) from three replicates. Statistical significance (*, *p* < 0.05; ***, *p* < 0.001) was determined by 1-way ANOVA with post hoc Dunnett’s test.

To determine whether PCAIs can inhibit 3D spheroid growth and/or induce apoptosis, breast cancer cells were cultured into compact spheroids and treated for 48 h with varying concentrations of the PCAIs. In both cell lines, 10 µM of PCAIs disrupted the structural integrity of the spheroids within 48 h (Figure 7A,D). Furthermore, acridine orange/ethidium bromide (AO/EB) staining at 48 h post-PCAIs-treatment revealed significant cell death in the spheroids at 10 µM (Figure 7B,E). Evaluating the mean AO/EB intensity of the spheroids, concentration-dependent decreases could be observed in MDA-MB-468 and MDA-MB-231 by 42 ± 3.6 and 34 ± 2.3%, respectively (Figure 7C,F).

### 3.8. PCAIs-Induced Cell Death Is Not RSK3-Driven

**Figure 8:**
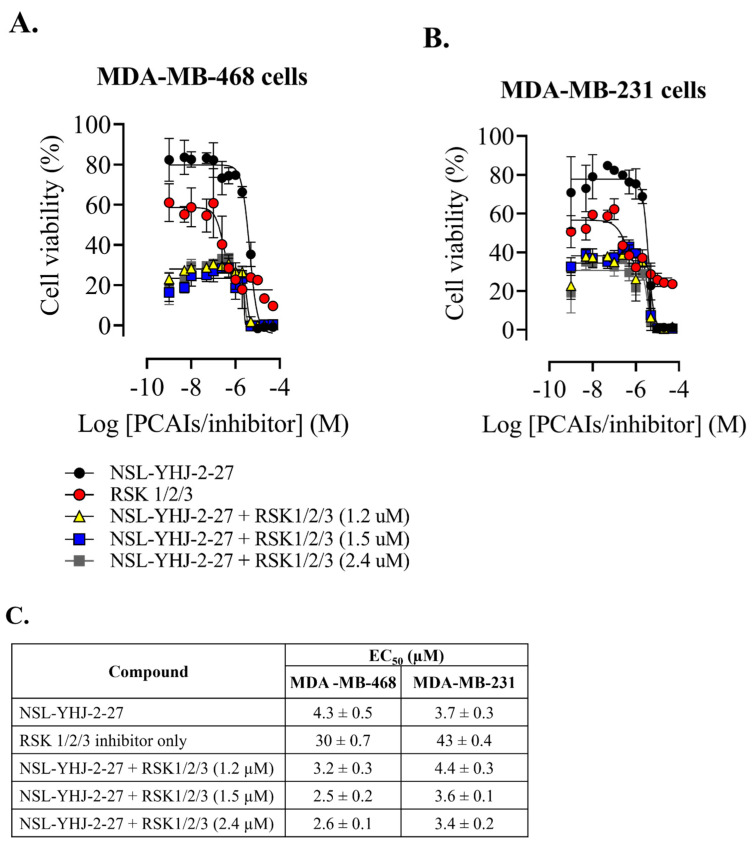
RSK1/2/3 inhibition does not reverse PCAIs-induced cell death. After treatment of cells with varying concentrations of NSL-YHJ-2-27 or RSK1/2/3 inhibitor (BRD7389) and varying concentrations of NSL-YHJ-2-27 in combination with the indicated concentrations of the RSK1/2/3 inhibitor for 48 h, resazurin reduction assay was performed (**A**,**B**) and EC_50_ value was calculated from non-linear regression (curve fit) using GraphPad Prism (**C**) from four replicates.

Previous studies have reported that p90RSK3 and -4 activation is pro-apoptotic. Our results show consistent increases in p90RSK phosphorylation in both MDA-MB-468 and MDA-MB-231 cells upon PCAIs treatment. We therefore wanted to know whether the PCAIs-induced cell death could be reversed using the p90RSK isoforms 1, 2, and 3 specific inhibitors. As shown in Figure 8A,B, the RSK 1/2/3 inhibitor did not prevent the PCAIs-induced death of MDA-MB-468 and MDA-MB-231 cells. Interestingly, when cells were exposed to both PCAIs and inhibitors, the EC_50_ improved in MDA-MB-468 and minimally in MDA-MB-231 cells. Here, we used BRD 7389, which is a ribosomal S6 kinase (RSK) inhibitor with IC_50_ values of 1.5, 2.4, and 1.2 μM for RSK1, RSK2, and RSK3, respectively [38]. The calculated EC_50_ values when PCAIs were combined with RSK1, RSK2, and RSK3 inhibition were 2.5 ± 0.2, 2.6 ± 0.1, and 3.2 ± 0.3, respectively, in MDA-MB-468. On the other hand, in MDA-MB-231, the EC_50_ values for PCAIs with inhibited RSK1, RSK2, and RSK3 enzymes were 3.6 ± 0.1, 3.4 ± 0.2, and 4.4 ± 0.3, respectively (Figure 8C). Although the results obtained did not particularly confirm that the cell death observed after PCAIs treatment was led by RSK-induced apoptosis, at least by RSK3, we believe that treatment with RSK inhibitors and PCAIs has potential in the development of combination therapies for breast cancer. These results do not preclude the possible involvement of RSK4 in the observed cells following NSL-YHJ-2-27 treatment.

### 3.9. PCAIs Inhibit Breast Cancer Cell Invasion into Matrigel

**Figure 9:**
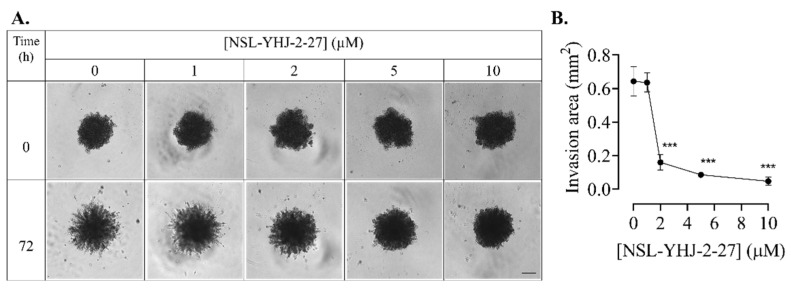
NSL-YHJ-2-27 inhibits breast cancer cell invasion into Matrigel. MDA-MB-231 cells were seeded in 96U round bottom Nunclon Sphera plates and allowed to grow for 48 h. The preformed spheroids were then treated with the indicated concentrations of NSL-YHJ-2-27 (0, 1, 2, and 5 µM), after which they were embedded in a Matrigel containing the corresponding concentrations of NSL-YHJ-2-27 in three replicates. Once the Matrigel solidified, brightfield (BF) images were obtained using a Nikon Ti Eclipse microscope at 4× magnification for 0 h exposure. (**A**) The spheroids were incubated for another 72 h, after which BF images were again obtained. Scale bar = 100 µm. (**B**) The area invaded by the cells was measured from the body of the spheroid using the NIS Element software. Statistical significance (***, *p* < 0.001) was determined by one-way ANOVA with post hoc Dunnett’s test.

Cancer cells are notorious for their ability to migrate into the circulation, invade new tissues, and establish secondary tumors, resulting in malignancy. To determine the ability of the PCAIs to inhibit invasion by breast cancer cells, compact spheroids were grown in Matrigel, and the area of invasion was measured 72 h after PCAIs treatment. Invading cells appeared as protruding extensions from the spheroid, with more prominent extensions occurring in the control spheroids and virtually none in those treated with 10 µM of NSL-YHJ-2-27 (Figure 9A). The invasion was inhibited by 74 ± 2.7, 87 ± 0.5 and 97 ± 1.8% in spheroids treated with 2, 5, and 10 µM of NSL-YHJ-2-27, respectively (Figure 9B). 

### 3.10. PCAIs Disrupt Actin Filaments and Focal Adhesion and Decrease Levels of Vinculin

**Figure 10:**
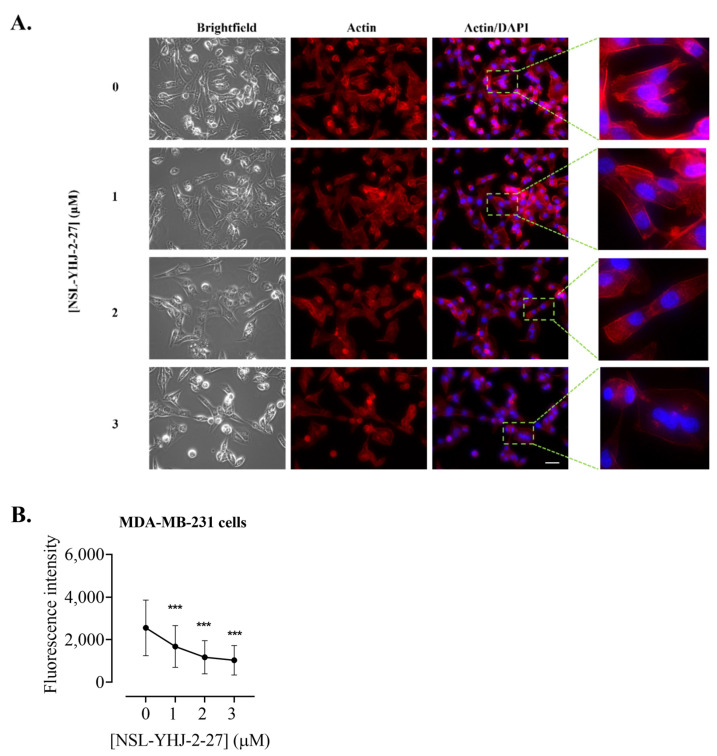
NSL-YHJ-2-27 disrupts F-actin filaments. Cells were grown in 8-well i*bidi* µ-slide until adherence. They were then treated with PCAIs for 48 h, followed by fixing and permeabilization. Actin filaments and nuclei were stained by incubating the cells in Alexa Fluor 568 Phalloidin and Hoechst, respectively. (**A**) Images were captured at 40× magnification using Keyence fluorescence microscope. Scale bar = 100 µm. (**B**) Total cellular fluorescence intensity was obtained by quantifying and averaging the intensities for *n* = 300 cells using Keyence BZ-X800 analyzer. Statistical significance (***, *p* < 0.001) was determined by one-way ANOVA with post hoc Dunnett’s test.

**Figure 11:**
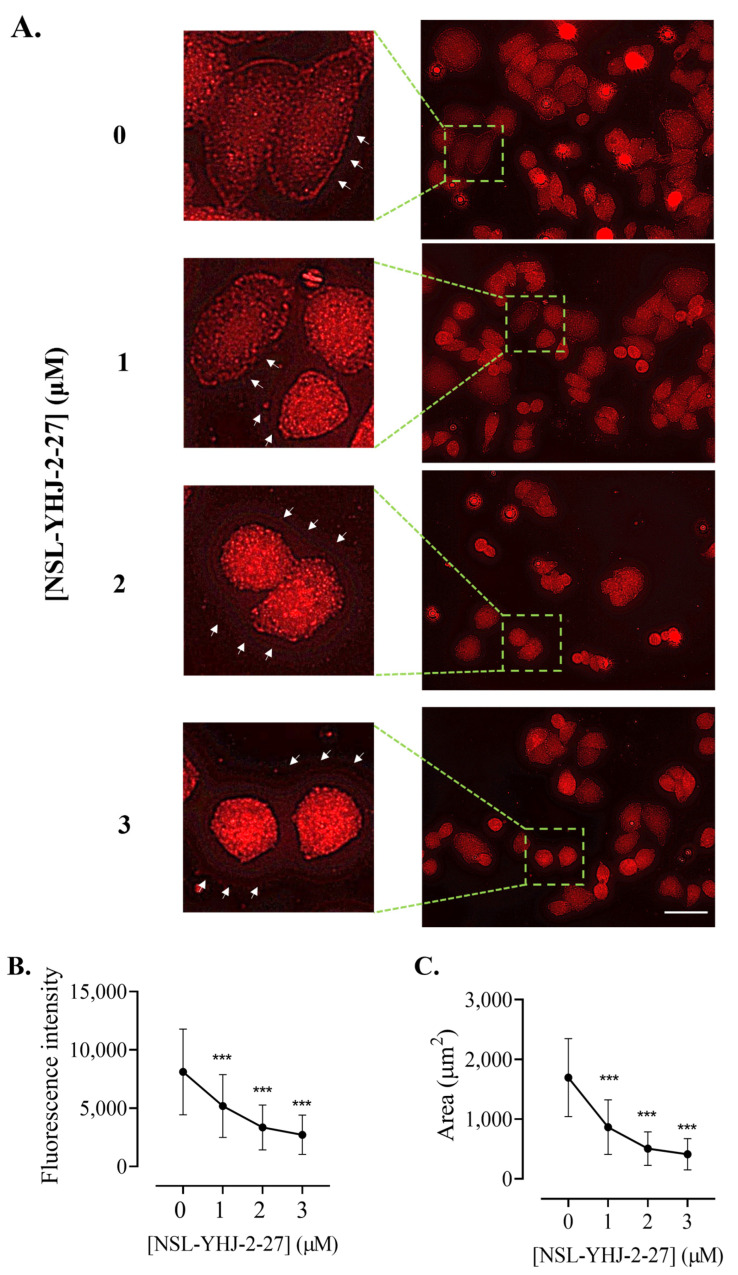
NSL-YHJ-2-27 disrupts the assembly of vinculin, leading to cell shrinkage. Cells were grown in 8-well i*bidi* µ-slides until adherence. They were then treated with PCAIs for 48 h and analyzed by immunocytochemistry for vinculin localization using anti-rabbit IgG Alexa Fluor 555 conjugate, as described in the Methods. Images were captured using Keyence fluorescence microscope at 40× magnification. Scale bar = 100 µm. (**A**). White arrows indicate the original ‘footprints’ of dislodged cells. In (**B**,**C**), *n* = 60 to 100 cells were quantified for fluorescence intensity and area using Keyence BZ-X800 analyzer. Data were plotted using GraphPad Prism version 8. Statistical significance (***, *p* < 0.001) was determined by one-way ANOVA with post hoc Dunnett’s test.

**Figure 12:**
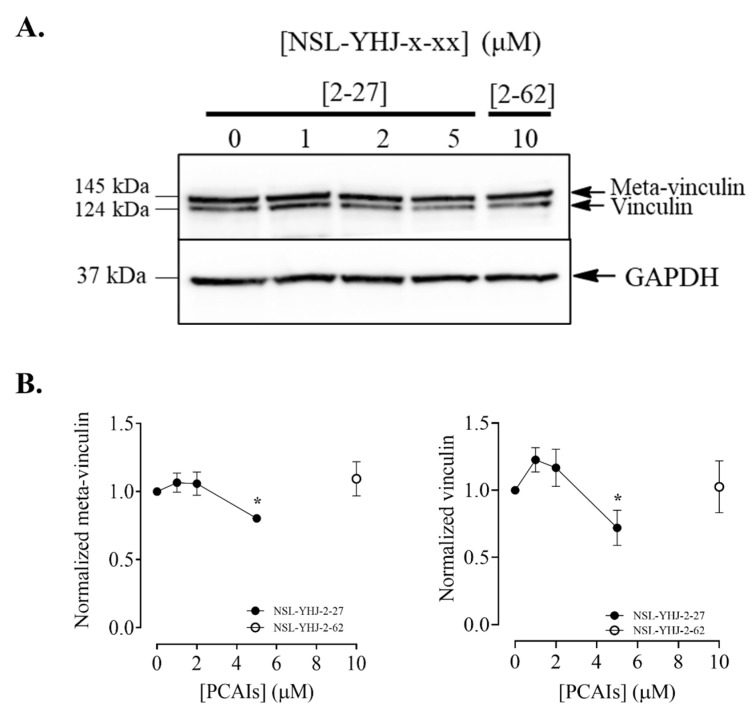
NSL-YHU-2-27 decreases the level of vinculin in MDA-MB-231 cells. Cells were treated for 48 h with the indicated concentrations of NSL-YHJ-2-27 or 10 μM of the non-farnesylated PCAIs analog NSL-YHJ-2-62. They were then lysed and analyzed by Western blotting for vinculin, as described in the Methods. (**A**) Western blot images and (**B**) chemiluminescence plots of bands following quantification using Image Lab software, normalized against GAPDH. Data are representative of three independent experiments. Statistical significance (*, *p* < 0.05) was determined by one-way ANOVA with post hoc Dunnett’s test.

The assembly and disassembly of F-actin are key factors for cellular processes such as adhesion, migration, and invasion, all of which are important for metastasis. To determine whether the PCAIs affected F-actin organization, MDA-MB-231 cells were exposed to NSL-YHJ-2-27 and then stained with Alexa Phalloidin 568, which binds to filamentous actin. Cells treated with the highest concentration of PCAIs exhibited diminished fluorescence as compared to the controls (Figure 10A). The fluorescence intensity in cells treated with 1, 2, and 3 µM decreased by 34, 54, and 60%, respectively (Figure 10B). Moreover, the effect of the PCAIs on focal adhesion was determined by immunocytochemical analysis for vinculin localization, since it is an adapter protein that interconnects actin and integrins. Control cells and those treated with 1 µM of NSL-YHJ-2-27 showed a defined outline of vinculin punctates spread all over the cytoplasm. Higher concentrations, however, resulted in rounded cells with the vinculin structures pushed up and compacted around the nucleus. Lightly fluorescent ‘footprints’ in areas from which the vinculin punctates were dislodged could be seen surrounding the shrunken cells (Figure 11A). Although rounded cells with more densely compacted punctates were obvious, the overall fluorescent intensities per cell for the shrunken cells were lower. The fluorescence intensity decreased by 66% and the area shrank by 76% after treatment with 3 µM of NSL-YHJ-2-27 (Figure 11B). The results also showed significant decreases in the levels of both vinculin and meta-vinculin (the integral membrane anchor protein for actin filaments) (Figure 12A) by 24 ± 2.2 and 39 ± 7.5%, respectively (Figure 11B).

## 4. Discussion

The major challenge faced in the management of TNBC is due largely to the lack of hormonal receptor positivity, for which targeted, more effective therapies are available. Novel drugs targeting TNBC are necessary to meet the therapeutic needs of TNBC patients. Previous work from our group revealed that polyisoprenylated methylated protein methyl esterase (PMPMEase) is overexpressed in pancreatic ductal adenocarcinoma and prostate cancers [32,35]. Moreover, the downregulation of the RAS-GAP RASA1 in a large percentage of breast cancer cases [42] and a small percentage of cases with KRAS mutations [8] suggests that RAS hyperactivity commonly drives breast cancer cases overall [43]. Furthermore, the disruption of mutant hyperactive growth factor receptor signaling at the level of KRAS in the MAP kinase pathway offers yet more options for breast cancer management. To this effect, PCAIs were developed to mitigate PMPMEase hyperactivity, which could act in combination with activating mutations and/or the overexpression of various monomeric G-proteins that cause or promote cancer. Hyperactive G-protein involvement in TNBC derives mainly from a reduction in RASA1 expression in up to 77% of TNBC cases [24,42]. The effects of PCAIs on the viability of cancer cell lines such as pancreatic [32,44], metastatic prostate [45], lung [33,37], and breast cancer [33,46] have previously been demonstrated and their anticancer effects predicted in view of the roles played by the G-proteins in cancer [33,36,37,46,47]. The PCAIs are modeled around the secondary modifications of G-proteins that are essential for protein–protein interactions [33]. Interactions with chaperone proteins such as calmodulin, galectins, and phosphodiesterase delta are crucial for intracellular trafficking and functional localization [48,49,50]. In fact, KRAS4B, a key oncoprotein, is trafficked to the inner surface of the plasma membrane by calmodulin [51]. Without this localization, the transduction of extracellular messages through the receptors to the MAP kinase and PI3K/AKT signaling pathways would be disrupted. Since the polyisoprenylated cysteine of the G-proteins is central to the interactions with the chaperone proteins, it was expected that the PCAIs may competitively disrupt the protein–protein interactions, thereby suppressing signal transduction and the MAP kinase and PI3K/AKT signaling pathways. The activation of the pathways by the PCAIs appears to suggest possible agonist actions through interactions with downstream RAS effectors such as RAF. This is possible as the interaction of G-proteins such as RAS with the RAF kinases involves interactions with the C-terminal polyisoprenylated cysteine [52]. Alternatively, it may be that the dislodgement of the G-proteins from the chaperone proteins increases the floating free intracellular pool of the G-proteins to spur the MAP kinase pathway’s activation. The disruption of the G-protein trafficking may also alter their usual interactomes and thus the signaling patterns and phenotypic outcomes. In our previous work [46], we observed the PCAIs-induced depletion of KRAS, RHOA, RAC1, and CDC42, which have a single polyisoprenyl group but are not lipid-modified, but which have the modifications mimicked by the PCAIs. On the contrary, those G-proteins, such as RAB5A, HRAS, and NRAS, that are modified either by two polyisoprenyl groups or a polyisoprenyl group and lipid thioester were relatively unaffected in lung cancer A549 and NCI-H1299 cells. In breast cancer MDA-MB-468 cells, HRAS and NRAS were depleted, and in MDA-MB-231 cells, only NRAS was depleted. These various effects may reflect specific molecular differences between the cell lines, such as thioesterases, which would hydrolyze the lipid modifications of the HRAS and NRAS proteins, leaving them modified only by the polyisoprenyl moiety [46].

It is apparent, therefore, that the unique cellular effects and the number of affected G-proteins with diverse pathway inputs add to the complexity of the observed molecular responses that defy the expected phenotypic effects typically associated with MAPK pathway stimulation. This pathway plays integral roles in several cell functions, such as growth, proliferation, apoptosis, and migration [53]. However, activating mutations in the genes encoding the RAS-RAF-MEK-ERK (RAS-MAPK) signaling cascade and the abnormal activation of receptor tyrosine kinases (RTKs) play key oncogenesis roles [54]. Remarkable increases in the levels of phosphorylated BRAF were observed in both cell lines after PCAIs treatment, while a decrease in the level of active CRAF was observed. Our results are in agreement with previous studies showing that the activation of BRAF promotes senescence and/or apoptosis [55], whereas the silencing of CRAF in melanoma cells induced apoptosis [56]. The RAF isoforms have been known to play distinct roles, as suggested by different knockout studies, particularly CRAF knockout studies, revealing that it is not necessary in the activation of the MAPK pathway, but, in the presence of BRAF, MAPK signaling can still occur [57], as observed in this study. The evaluation of downstream MAPK pathway kinases reveals results that concur with previous findings obtained from a different cell line (lung cancer cell, A549) [33]. The activation of the MEK 1/2, ERK 1/2, and p90RSK is prominent after PCAIs treatment. Although the phosphorylation of these enzymes, particularly ERK 1/2, indicates MAPK pathway activation, which typically results in increases in cell proliferation and survival, several studies have shown that ERK activation sometimes results in programmed cell death depending on the stimuli, duration, intensity of stimulation, and cell types [58,59]. Specifically, prolonged stimulation appears to initiate pro-apoptotic activity [60,61]. Overactivation of the RAS/ERK signaling pathway can effectively lead to senescence, cell-cycle arrest, and/or apoptosis [62,63]. On a more specific note, a study on a neuroblastoma cell line revealed that increased ERK pathway signaling leads to enhanced apoptosis after anthracycline treatment [64]. Furthermore, the phosphorylation of p90RSK is also associated with the activation of its isoforms, RSK 1 to 4 [21]. Available evidence suggests that some of the RSK isoforms perform strikingly different biological roles. For example, RSK-1 and RSK-2 promote cancer cell growth, survival, and proliferation, whereas RSK-3 and RSK-4 are reported to initiate cell arrest and apoptosis and perform functions similar to tumor suppressors [21,22,65]. The mechanisms by which RSK-3 and RSK-4 inhibit cell proliferation are still unknown, but studies on these isoforms reveal that they induce a G1-phase arrest, as well as apoptosis, when overexpressed in ovarian and breast cancer cells [66,67]. In another study on breast tumors, an increase in the expression of phosphorylated p90RSK showed a proportional tumor size reduction by 12% during neoadjuvant chemotherapy, as measured by magnetic resonance imaging [68]. Our results showing increased p90RSK phosphorylation levels in both TNBC cell lines are in agreement with these previous studies, showing that the activation of p90RSK can indeed inhibit cancer cell growth and proliferation. Although complicated by the effects of the RSK 1/2/3 inhibitors that cause cell death, p90RSK phosphorylation that accompanies cell death suggests that RSK-3 and RSK-4 are most likely responsible. Future work to identify the specific isoform responsible will involve immunoaffinity precipitation and mass spectrometric analysis of the protease digest. Another approach would involve the specific knockdown of the different isoforms in addition to the PCAIs treatments. Although AKT signaling generally promotes proliferation and survival, it may also promote cell death in certain circumstances. Another important finding in this study is the activation of AKT in MDA-MB-468 cells. AKT activation leads to an increase in oxidative stress and consequent reactive oxygen species (ROS)-triggered cell death [69]. The opposite effect of the PCAIs-induced inhibition of AKT phosphorylation in MDA-MB-231 cells, a *KRAS*-mutant breast cancer, is interesting. It is difficult to know whether the KRAS mutation or the lack of it is responsible for the observed opposing effects of PCAIs on AKT phosphorylation between the two breast cancer cell lines. Further analysis using more cell lines with and without KRAS mutations will help to establish the role of hyperactive KRAS in the response of cancer cells to PCAIs’ effects on AKT phosphorylation. Another approach will be to use specific inhibitors of AKT to determine whether AKT inhibition in the cell lines in which it is activated in response to PCAIs treatment will result in the reversal of the phenotypic response of cell death. What is certain now is that KRAS signaling feeds into the PI3K/AKT pathway, implying that AKT may already be hyperactivated in cells with hyperactive KRAS signaling. The observed activation of MAPK kinases points to the promotion of apoptosis, which further explains the potency of PCAIs against breast cancer cells. This agrees with the flow cytometry results, wherein higher concentrations of PCAIs shifted the population of viable cells to the early and late apoptosis stages. Moreover, an increase in the pro-apoptotic protein BAK1 and a decrease in full-length or noncleaved Caspases 3 and 7 were observed, indicating the initiation of apoptosis after PCAIs treatment.

**Figure 13:**
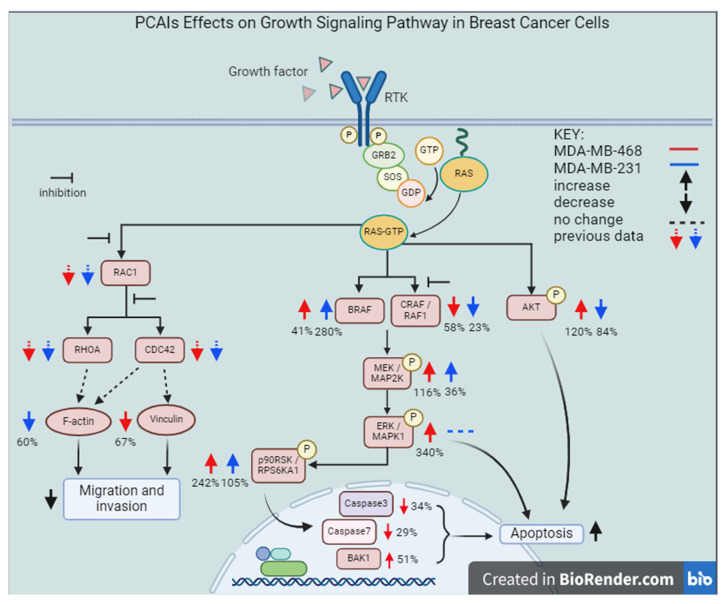
Proposed mechanism of action of the PCAIs in breast cancer cell lines. Abbreviations: RTK, Receptor Tyrosine Kinase; GRB2, Growth Factor Receptor Bound Protein 2; SOS, Son-of-Sevenless; GDP, Guanosine Diphosphate; GTP, Guanosine Triphosphate; RAS, Rat Sarcoma; BRAF, Rapidly Accelerated Fibrosarcoma, v-Raf Murine Sarcoma Viral Oncogene Homolog B; CRAF, RAF Proto-Oncogene Serine/Threonine-Protein Kinase; MEK, Mitogen-Activated Protein Kinase Kinase; ERK, Extracellular-Signal-Regulated Kinases; p90RSK, 90 kDa Ribosomal s6 Kinases; AKT, Protein Kinase B; BAK1, BCL2 Antagonist/Killer 1.

A key feature of G-protein action in cancer progression is their role in cytoskeleton formation and focal adhesion, both of which are essential for cell migration and invasion in metastasis as well as angiogenesis [70]. Our results revealing that PCAIs inhibit the invasion of cells, likely due to the disruption of cytoskeletal proteins, are in agreement with the widely reported roles of G-proteins in cytoskeletal function. Since cell movement is facilitated by the reorganization of the actin cytoskeleton involving actin polymerization and disassembly [71], changes in F-actin levels result in disorganized actin filaments. For cell migration to occur, there must be coordination between the actin cytoskeleton and focal adhesion. The finding in the present study that exposure to PCAIs causes actin filament disruption, resulting in lower levels of actin filaments in cells, means a loss of cell integrity and motility. Moreover, depleted vinculin levels and cell retraction from vinculin punctates in the periphery, leaving behind vinculin punctate ‘footprints’, clearly demonstrate the disruption of focal adhesion, which explains the cell rounding resulting from PCAIs treatment. These observations corroborate our previous study, in which the exposure of non-small-cell lung cancer (NSCLC) to PCAIs resulted in suppressed migration and invasion in both 2D and 3D cultures, changes in morphological features that led to cell rounding, disrupted F-actin organization and reduced filopodia density, and depleted levels of the RHO family of proteins, RAC1, CDC42, and RHOA [47]. Other than changes in the levels of RHO family proteins, which are known to be the drivers of metastatic lung cancer [72], the PCAIs also reduced the levels of full-length integrin α4, vinculin, and Rock1, abrogating cytoskeleton remodeling, which led to the suppression of focal adhesion formation [36]. Moreover, the levels of vinculin and fascin were also significantly reduced in A549 cells after PCAIs exposure [46]. In addition to NSCLC, depleted levels of RAC1, CDC42, and RHOA were also observed in several other cell lines, including lung cancer (A549 and NCI-H1299) and breast cancer (MDA-MB-468 and MDA-MB-231) [46], following PCAIs treatment. We believe that the observed changes in the morphology and impeded motility of the breast cancer cells after exposure to PCAIs are due to the disrupted F-actin organization, depleted levels of RHO GTPases, and vinculin punctate disruption. Overall, the present data suggest the possible mechanism of action of the PCAIs as summarized in Figure 13. The potency of the PCAIs to inhibit the viability, growth, and movement of breast cancer cells has been comprehensively examined in this study. In addition to questions posed by the current data, such as a better understanding of the disparate effects of the PCAIs on AKT phosphorylation in the two cell lines and the identification of the specific p90RSK that is phosphorylated in response to the PCAIs, future work will also involve the identification of the direct pharmacological target and evaluation of the agents in tumor-derived organoids and in an in vivo model of breast cancer.

## 5. Conclusions

The PCAIs treatment of breast cancer cell lines abrogates cancer progression processes that are essential for proliferation, migration, and invasion, which are mediated by G-proteins. These findings hold promise for the further development of targeted therapies to treat breast cancer cases with hyperactive KRAS and corresponding upstream hyperactive receptors. 

## Figures and Tables

**Table 1:** PCAIs structure. The structure of the experimental PCAIs, NSL-YHJ-2-27, and control PCAIs analog without the polyisoprenyl moiety NSL-YHJ-2-62.

Compound	Structure
NSL-YHJ-2-27	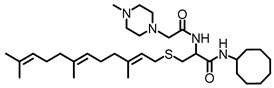
NSL-YHJ-2-62	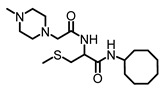

## Data Availability

The data reported in this manuscript has all been provided in the manuscript.
